# A *de novo *marker chromosome derived from 9p in a patient with 9p partial duplication syndrome and autism features: genotype-phenotype correlation

**DOI:** 10.1186/1471-2350-11-135

**Published:** 2010-09-21

**Authors:** Khaled K Abu-Amero, Ali M Hellani, Mustafa A Salih, Mohammad Z Seidahmed, Tageldin S Elmalik, Ghassan Zidan, Thomas M Bosley

**Affiliations:** 1 the Ophthalmic Genetics Laboratory, Department of Ophthalmology, College of Medicine, King Saud University, Riyadh, Saudi Arabia; 2 the PGD Laboratory, Saad Specialist Hospital, Al-Khobar, Saudi Arabia; 3 the Department of Pediatrics (Division of Pediatric Neurology), College of Medicine, King Saud University, Riyadh, Saudi Arabia; 4 Department of Pediatrics, Security Forces Hospital, Riyadh, Saudi Arabia; 5 Psychiatry Department, Security Forces Hospital, Riyadh, Saudi Arabia; 6 Department of Pathology (Cytogenetics Laboratory), King Khalid University Hospital, Riyadh, Saudi Arabia; 7 the Division of Neurology, Cooper University Hospital, Camden, NJ, USA

## Abstract

**Background:**

Previous studies focusing on candidate genes and chromosomal regions identified several copy number variations (CNVs) associated with increased risk of autism or autism spectrum disorders (ASD).

**Case Presentation:**

We describe a 17-year-old girl with autism, severe mental retardation, epilepsy, and partial 9p duplication syndrome features in whom GTG-banded chromosome analysis revealed a female karyotype with a marker chromosome in 69% of analyzed metaphases. Array CGH analysis showed that the marker chromosome originated from 9p24.3 to 9p13.1 with a gain of 38.9 Mb. This mosaic 9p duplication was detected only in the proband and not in the parents, her four unaffected siblings, or 258 ethnic controls. Apart from the marker chromosome, no other copy number variations (CNVs) were detected in the patient or her family. Detailed analysis of the duplicated region revealed: i) an area extending from 9p22.3 to 9p22.2 that was previously identified as a critical region for the 9p duplication syndrome; ii) a region extending from 9p22.1 to 9p13.1 that was previously reported to be duplicated in a normal individual; and iii) a potential ASD locus extending from 9p24.3 to 9p23. The ASD candidate locus contained 34 genes that may contribute to the autistic features in this patient.

**Conclusion:**

We identified a potential ASD locus (9p24.3 to 9p23) that may encompass gene(s) contributing to autism or ASD.

## Background

Autism spectrum disorders (ASD), including autism, are neuro-developmental disorders characterized by impairment in social and communication skills together with stereotyped and repetitive behavior and/or a restricted range of interests. Current prevalence estimates in the United States are 0.1-0.2% of live births for autism and 0.6% for ASD [1]. The exact prevalence of ASD in Saudi Arabia is still undetermined, but one rough estimate is 18 per 10,000, slightly higher than 13 per 10,000 reported in developed countries [2]. Reasons for a possibly higher prevalence of ASD in Saudi Arabia are not clear but could relate in part to differences in diagnostic practice, higher consanguinity rates, a founder effect, or unidentified environmental risk factors.

Previous studies focusing on candidate genes and chromosomal regions identified several copy number variations (CNVs) associated with increased risk of ASD [3-9]. Chromosomal regions implicated by these studies include 1q, 1p, 5q, 7q, 15q, 16p, 17p, 20p, 3p, 10q, 15q, 20p, 22q and Xq with patients exhibiting variable expressivity and associated phenotypes such as schizophrenia, mental retardation, developmental delay, and epilepsy [10]. The Autism Chromosome Rearrangement Database (http://projects.tcag.ca/autism) currently lists 15 reports of autistic patients with chromosomal anomalies involving 9p. Eleven of these cases involved CNVs of 9p together with other chromosomes, while four cases involved an isolated 9p deletion or inversion. We describe a 17-year-old girl with autism, mental retardation, and epilepsy with features of the 9p duplication syndrome who has a mosaic marker chromosome derived from 9p24.3 to 9p13.1.

### Case Presentation

**Figure 1:**
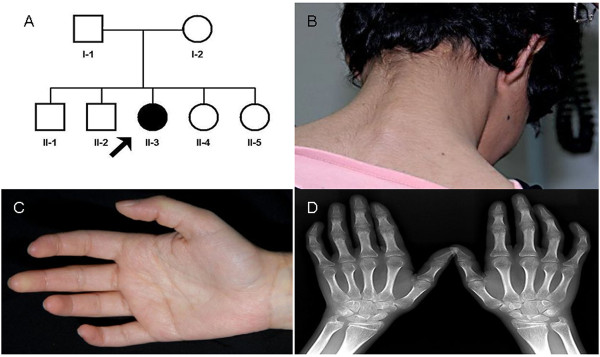
**Family pedigree and dysmophic features observed**. **A**) Family pedigree identifying the proband as II-3. Dysmorphic features found in the proband included **B**) webbing of the neck and **C**) fifth finger clinodactyly. **D**) X-ray of hands and wrists showed evidence of osteoporosis and clinodactyly.

The proband (II-3, Figure 1A) was first examined at age 15 months because of seizures and developmental delay and has been followed since then by MAS and MZS. She has been institutionalized for most of her life. Her parents were unrelated Saudi Arabs, and she had four unaffected siblings, although the family history included epilepsy and cerebral palsy in maternal cousins. Pregnancy was unremarkable, delivery was normal at term, and she had no neonatal problems. This study was performed in accordance with the regulations of the King Saud University College of Medicine Ethics Committee (approval # E-09-010), and the proband's legal guardian signed informed consent. The study adhered to the tenets of the Declaration of Helsinki.

At the age of 10 months, she developed flexion myoclonic jerks with hypsarrhythmia on EEG typical of infantile spasms, and she was treated with a course of prednisone (2 mg/kg) and clonazepam. A repeat EEG was moderately abnormal with multifocal epileptic discharges consistent with secondary symptomatic epilepsy, and she was started on the anticonvulsants vigabatrin and clonazepam and on risperidone for autistic behavior. Vigabatrin was successfully withdrawn and replaced by lamotrigine with no seizures after the age of 3 ½ years. She had delayed motor and cognitive functions, sitting at 13 months and walking at 2 years. She never developed speech, and she was diagnosed as severely mentally retarded with autistic features at age 6 years with a Vineland social maturity scale score of 31. She was unable to feed herself and was incontinent of both stool and urine.

Laboratory investigation revealed normal hematologic indices, liver function, and electrolytes. Tandem mass spectrometry (TMS) for metabolic disorders was unremarkable. MRI of the brain and brainstem auditory evoked responses were both normal.

On examination, she had growth retardation with reduced weight (28.3 kg; <2 SD below the mean), height (137 cm; < 2 SD), and head circumference (49 cm; < 3 SD). She had subtle dysmorphic features consistent with a partial 9p duplication syndrome with deep set eyes, small upper lip, webbing of the neck (Figure 1B) and clinodactyly (Figure 1C). X-rays of hands and wrists revealed normal bone age with osteoporosis and confirmed clinodactyly of the fifth finger (Figure 1D). She had no skin hypopigmentation or other stigmata of neurocutaneous disorders, but the dorsum of the left hand and the bridge of her nose were hyperpigmented because of self injury.

On neurologic examination, she was awake and alert but unable to follow commands or speak other than some echolalia and repetitive non-specific sounds. She moved all four extremities with normal tone; reflexes were brisk, but toes were down going. Ophthalmologic exam and ocular motility were grossly normal, but she did not make eye contact. She responded appropriately to visual threat and loud sounds. She was generally quiet and motionless, typically leaning to one side in an awkward posture, making stereotyped movements of her hands and trunk, and frequently hitting her nose with her hand and grinding her teeth. Social interaction was minimal with no smile, response to commands, or copying of examiner's movements. Applying DSM4-R criteria and PDD assessment scale/screening questionnaire, a psychiatric assessment yielded the diagnoses of (Axis I) pervasive developmental disorder (autistic type) and (Axis II) severe mental retardation.

Conventional cytogenetic analysis on GTG-banded chromosomes was performed according to the standard technique on cultured lymphocytes from the father (I-1), the mother (I-2), the proband (II-3), and her unaffected brother (II-1) after obtaining informed consent. GTG-banded chromosomes analysis was not carried out on individuals II-2, II-4 and II-5 (see Pedigree Figure 1A).

Agilent human CGH microarrays (Agilent Technologies, Santa Clara, CA, USA) were employed on individuals I-1, I-2, II-1, II-2, II-3, II-4, and II-5 using chips containing unique oligonucleotides for 244,000 probes (244 K) with average probe spacing across the human genome of 6.4 Kb. Labeling reactions were performed with 1 μg genomic DNA with Agilent Genomic DNA Labeling Kit PLUS (Agilent Technologies) according to manufacturer's protocol, and the microarray chip was then scanned by the Agilent Microarray Scanner.

Data analysis was performed by Agilent Feature Extraction 9.1 and CGH Analytics 3.4. In brief, log2 expression ratios were computed and normalized for forward and reverse fluor (i.e. dye-swap) experiments using the CGH Analytics 3.4 software. Putative chromosome copy number changes were defined by intervals of three or more adjacent probes with log2 ratios suggestive of a deletion or duplication when compared with the log2 ratios of adjacent probes. The quality-weighted interval score algorithm (ADM2) was used to compute and assist in the identification of aberrations for a given sample. Controls were 258 normal individuals of similar ethnic background. Genetic databases employed included Genbank (http://www.ncbi.nlm.nih.gov/genbank/), GeneCards (http://www.genecards.org/), the Autism Chromosome Rearrangement Database (http://projects.tcag.ca/autism), OMIM (http://www.ncbi.nlm.nih.gov/sites/entrez?db=omim) and literature search on PubMed (http://www.ncbi.nlm.nih.gov/pubmed).

**Figure 2:**
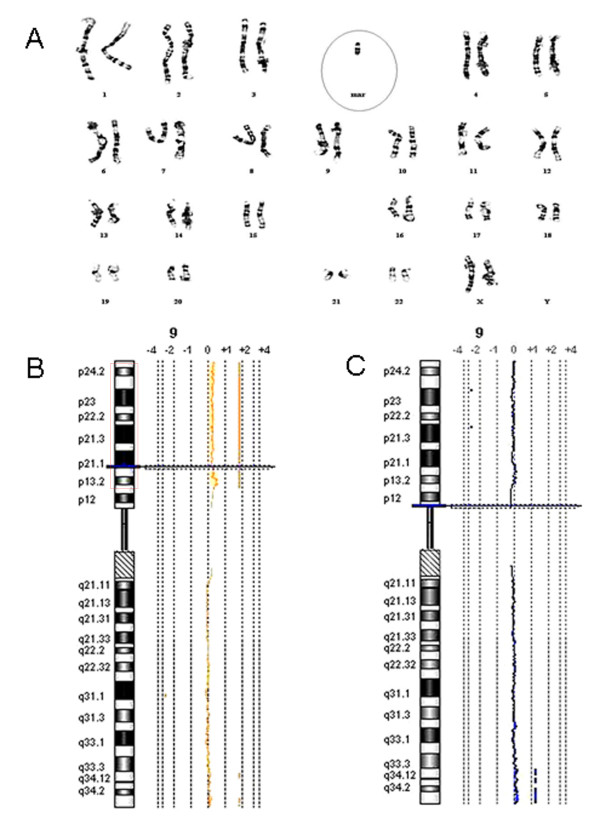
**GTG-banded Karyotype and array CGH results**. **A**) GTG-banded karyotype showing the marker chromosome (circled). **B**) Array CGH demonstrating a chromosome 9p duplicated region in the proband (duplicated area is boxed). **C) **Array CGH results in parents, healthy siblings, and controls were normal. Array CGH for individual II-2 is shown.

Conventional cytogenetic analysis on GTG-banded chromosomes revealed a female karyotype in the proband with a marker chromosome (Figure 2A) in 69% of the analyzed metaphases after counting more than 150 metaphase spreads; thus, her karyotype was 47,XX,+mar[69]/46,XX[31]. The marker chromosome was relatively small and was found in the proband but not in her parents or unaffected sibling (II-1), all of whom had a normal karyotype. No other chromosomal abnormalities were detected in the patient, or her parents, or her tested unaffected sibling (II-1).

Oligonucleotide whole genome array CGH analysis showed that the isolated marker chromosome originated from 9p. The duplicated area started from the region surrounding the *CBWD1 *locus and included 9p24.3 to 9p13.1 (from 153131 bp to 39131894 bp on chromosome 9) with a size of 38.9 Mb (Figure 2B). This gain of chromosome 9p was detected only in the proband. Her parents, unaffected siblings (II-1, II-2, II-4 and II-5), and 258 ethnically matched controls all had normal array CGH results (Figure 2C). No other chromosomal abnormality was detected in any individual examined by array CGH.

**Figure 3:**
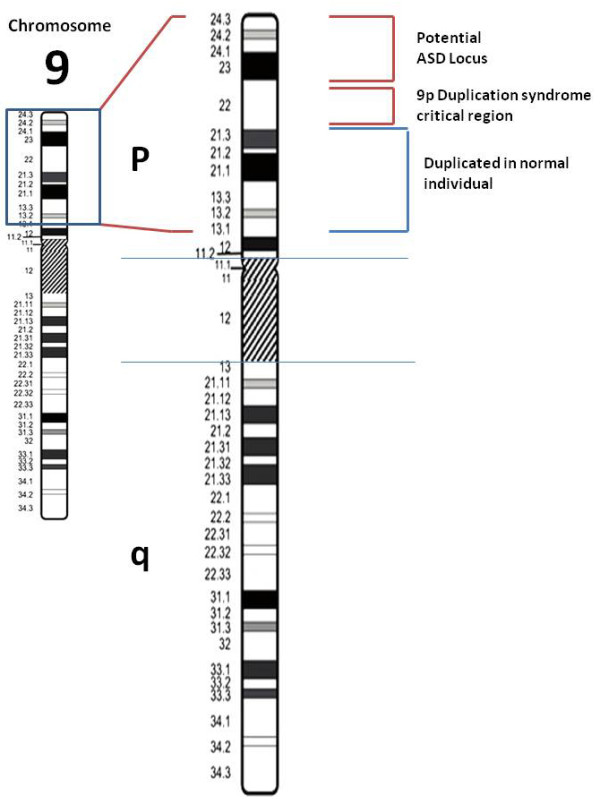
**Ideogram showing the duplicated area**. Chromosome 9 ideogram illustrating (to left, boxed) the overall duplicated area and (to right, labeled) the duplicated area in a normal individual, the 9p duplication syndrome critical region, and the potential ASD locus.

The NCBI Map Viewer revealed that the duplicated area encompassed 381 genes (Additional file 1: **Table S1**). Analysis of the duplicated area identified: i) an area extending from 9p22.3 to 9p22.2 previously identified as critical for the 9p duplication syndrome [11]; ii) a region extending from 9p22.1 to 9p13.1 previously reported to be duplicated in a girl with minimal physical findings and normal IQ [11] and iii) a potential ASD locus extending from 9p24.3 to 9p23 (Figure 3). This potential ASD locus is contained within the larger area identified by Szatmari and colleagues (9p24.3 to 9p13.2) in their study of 33 CNV gains in patients with autism [5].

The first patient with trisomy 9p was described by Rethore et al. in 1970 [12], and more than 150 patients with partial or complete 9p trisomy have been reported since then [13]. In most patients, the 9p trisomic segment was derived from a parent carrying a reciprocal balanced translocation and was accompanied by a concurrent deletion of another chromosome. Isolated *de novo *duplications of 9p without a concurrent deletion are infrequent, with 15 patients reported to date [11,14-26]. Typical characteristics include growth and mental retardation, microbrachycephaly, deep and wide-set eyes with down-slanting palpebral fissures, prominent nasal root with a bulbous nasal tip, down-turned corners of the mouth, low-set ears, short fingers and toes with hypoplastic nails, and delayed bone age. Clinodactyly has also been reported in a patient with trisomy 9p and insertion on chromosome 12 [27]. Our patient had only partial 9p duplication features despite encompassing the entire 9p duplication critical region (D9S162-D9S267; 9p22.3 to 9p22.2) in the duplication, and this may be because of the mosaic nature of the duplication.

One landmark study [28] provided evidence that chromosomal mosaicism plays an important role in the generation of meiotic aneuploidy, which in turn is recognized as a leading cause of human prenatal death, congenital malformations, and learning disabilities [29]. The largest study to date on mosaicism in autistic boys found mosaic aneuploidy in 19 of 116 children (16%) with idiopathic autism [30]. The degree of mosaicism in our patient (69%) may imply that gene dosage of 9p is a factor contributing to autistic features; however, this remains unproven because other tissues, including brain, were not available.

We describe a 17-year-old Saudi girl with autism associated with mental retardation and seizures despite a non-focal neurologic examination and unremarkable neuroimaging [31]. Autism has not been associated previously with an isolated 9p duplication [13], but this patient had a large, isolated, mosaic 9p24.3 to 9p13.1 duplication that is likely symptomatic given that the duplication was *de novo*, that it was not a polymorphic variant, that it segregated with autism in this family, and that some genes in the duplicated region have been linked previously to autistic features.

She had a potential ASD locus extending from 9p24.3 to 9p23 (Figure 3) which, after removing hypothetical genes, encompassed 34 genes (Additional file 2: **Table S2**). The role of 16 of these genes in autism could not be assessed because they have unknown function using available databases and literature (see Methods). Of the 18 genes with known function, five (*FOXD4L4*, *FOXD44L2*, *FOXD4*, *FOXD4L1 *and *FOXD2*, all in the forkhead box gene family) are involved in transcription regulation. The very low density lipoprotein receptor gene (*VLDLR*) belongs to a well-known family of similar genes that have the ability to transduce a diversity of extracellular signals across neuronal membranes in the adult central nervous system. Their role in modulating synaptic plasticity and their necessity in hippocampus-specific learning and memory are well documented [32]. Szatmari et al [5] and others [33-36] suggested that the genes *DOCK8*, *DMRT1*, *DMRT3*, and *DMRT2 *from this area of 9p may contribute to the autistic spectrum disorder phenotype [35]. The remaining eight genes in this locus (*KANK1*, *EIF1*, *LOC642350*, *BHLHB3*, *SMARCA2*, *KCNV2*, *GPS2 *and *ATP5H*) are involved in biological processes such as regulation of actin polymerization, nucleic acid binding, nucleotide and chromatin organization, mediating voltage-dependent potassium ion permeability, intracellular signaling, and ATP synthesis. No conclusion could be reached at this time about whether they may be involved in autism or ASD.

## Conclusion

To our knowledge, this is the first report of an autistic patient with an isolated mosaic *de novo *9p duplication. Analysis of the duplicated area identified a potential ASD locus 9p24.3 to 9p23, implying that screening this area for copy number variations by array CGH may help identify specific gene(s) contributing to autism or ASD.

## Abbreviations

Array CGH: Array comparative genomic hybridization; ASD: Autism spectrum disorders; CNVs: Copy number variations; DSM: Diagnostic and statistical manual of mental disorders; EEG: Electroencephalogram; mar: marker; MRI: Magnetic resonance imaging; PCR: Polymerase chain reaction; PDD: Pervasive developmental disorders; SD: Standard deviation; TMS: Tandem mass spectrometry

## Competing interests

The authors declare that they have no competing interests.

## Authors' contributions

KKA was in charge of study design, analysis of genetic data, and writing the genetic part of the manuscript. AH carried out array CGH studies. MAS, MZS, TSE and TMB were all essential in clinical evaluation of the patient and compiling the clinical data in the manuscript. GZ carried out the karyotyping. TMB was in charge of the overall project design, assessing clinical and genetic data, and writing the manuscript. All authors read and approved the final manuscript.

## Pre-publication history

The pre-publication history for this paper can be accessed here:

http://www.biomedcentral.com/1471-2350/11/135/prepub

## Supplementary Material

Additional file 1 **Supplementary Table S1**. Genes encompassed in the duplicated area. Click here for file

Additional file 2 **Supplementary Table S2**. Potentially important genes in the duplicated area. Click here for file
